# Differential Effects of Phosphatidylinositol 4-Kinase (PI4K) and 3-Kinase (PI3K) Inhibitors on Stomatal Responses to Environmental Signals

**DOI:** 10.3389/fpls.2017.00677

**Published:** 2017-05-01

**Authors:** Sho Takahashi, Keina Monda, Takumi Higaki, Mimi Hashimoto-Sugimoto, Juntaro Negi, Seiichiro Hasezawa, Koh Iba

**Affiliations:** ^1^Department of Biology, Faculty of Science, Kyushu UniversityFukuoka, Japan; ^2^Department of Integrated Biosciences, Graduate School of Frontier Sciences, The University of TokyoChiba, Japan; ^3^Graduate School of Bioagricultural Sciences, Nagoya UniversityNagoya, Japan

**Keywords:** *Arabidopsis thaliana*, phosphatidylinositol 3-kinase (PI3K), phosphatidylinositol 4-kinase (PI4K), PATROL1, stomata

## Abstract

Specific cellular components including products of phosphatidylinositol (PI) metabolism play an important role as signaling molecules in stomatal responses to environmental signals. In this study, pharmacological inhibitors of a set of cellular components, including PI4-kinase (PI4K) and PI3K, were used to investigate stomatal closure in response to CO_2_, darkness, and abscisic acid (ABA). Treatment with PAO, a specific inhibitor of PI4K, specifically inhibited the stomatal response to CO_2_ compared with that to darkness and ABA. In contrast, treatment with LY294002, a PI3K-specific inhibitor, specifically inhibited the stomatal response to darkness compared with that to CO_2_ and ABA. The specific inhibitory effects of PAO and LY294002 were also observed as changes in the spatial density of dot-like structures labeled by green fluorescent protein-tagged PATROL1, a protein that controls stomatal aperture possibly via regulation of H^+^-ATPase amount in guard cell plasma membranes. Our results suggest an important role for PI4K and PI3K in the CO_2_ and darkness signal transduction pathways, respectively, that mediate PATROL1 dynamics.

## Introduction

Stomata, formed by a pair of guard cells, have developed the ability to respond to various environmental signals (Schroedar et al., 2001). Environmental signals such as darkness or CO_2_ concentration and a plant hormone abscisic acid (ABA) are major factors triggering the stomatal closure response. The signals perceived by stomata activate intracellular molecules linked to a variety of signaling molecules (Kollist et al., 2014). Previous studies on ABA signaling showed that the stomatal response is mediated by numerous cellular components. For example, phosphatidylinositol (PI) metabolism plays an important role in ABA-induced oscillations in guard cell cytosolic Ca^2+^ concentration ([Ca^2+^]_cyt_) (Staxen et al., 1999). PI 3-phosphate (PI3P) and PI 4-phosphate (PI4P) are required for normal stomatal movements and are involved in the ABA-induced increase in [Ca^2+^]_cyt_ (Jung et al., 2002). cGMP pathways are involved in the increase in [Ca^2+^]_cyt_ by promoting Ca^2+^ release from intracellular stores (Garcia-Mata et al., 2003). Nitric oxide (NO) synthesis is required for ABA-induced stomatal closure (Neill et al., 2002). Microtubule organization is also essential for stomatal movement (Marcus et al., 2001; Jiang et al., 2014). Furthermore, the SNARE complex controls guard cell ion channel activities (Leyman et al., 1999; Sutter et al., 2007; Eisenach et al., 2012). The mechanisms for environmental regulation of stomatal aperture are only now being elucidated because of the complex networks of signaling components involved (Assmann and Jegla, 2016). We hypothesize that components of the ABA signal transduction pathway may also be important for the environmental regulation of stomatal aperture.

The activity of the plasma membrane H^+^-ATPase that drives stomatal opening may be regulated downstream of the ABA signal transduction pathway. PATROL1, which was identified by a mutant impaired in stomatal opening in response to low CO_2_ and light, is hypothesized to deliver the H^+^-ATPase to plasma membranes by promoting the formation of SNARE complexes (Hashimoto-Sugimoto et al., 2013). Live cell imaging revealed that green fluorescent protein-tagged PATROL1 (GFP-PATROL1) is localized to dot-like structures adjacent to the plasma membranes in guard cells (Hashimoto-Sugimoto et al., 2013). The spatial density of the GFP-PATROL1 dots increased in guard cells with closed stomata treated with darkness or drought stress, suggesting downregulation of H^+^-ATPase activity by the PATROL1 internalization and a subsequent decrease in the amount of H^+^-ATPase in plasma membranes (Hashimoto-Sugimoto et al., 2013).

In this study, we performed a pharmacological study using inhibitors of a set of cellular components that included PI3K, PI4K, cGMP, Ca^2+^, small G-protein, the SNARE complex, and microtubules. We found that PAO (a PI4K inhibitor) and LY294002 (a PI3K inhibitor) specifically affected the stomatal response and PATROL1 dynamics in response to CO_2_ and darkness, respectively. These findings suggest the possibility that the products of PI metabolism including PI4P or PI3P selectively regulate stomatal movements depending on the environmental signals perceived in guard cells.

## Materials and Methods

### Plant Materials

*Arabidopsis thaliana* (ecotype Columbia-0) plants were grown on solid 1/2 MS medium for 18 days in a growth chamber (constant white light of 80 μmol m^-2^ s^-1^ at 22–28°C and 30–60% relative humidity) after being stored at 4°C in the dark for 2 days. The plants were transplanted onto a nutrient solution composed of the following macronutrients: 1.25 mM KNO_3_, 0.5 mM Ca(NO_3_)_2_, 0.5 mM MgSO_4_, 0.625 mM KH_2_PO_4_, and the following micronutrients: 17.5 μM H_3_BO_3_, 12.5 μM Fe EDTA 3H_2_O, 3.5 μM MnCl_2_ 4H_2_O, 2.5 μM NaCl, 0.25 μM ZnSO_4_ 7H_2_O, 0.125 μM CuSO_4_ 5H_2_O, 0.05 μM Na_2_Mo_4_ 2H_2_O, and 0.0025 μM CoCl_2_ 6H_2_O. The final solution pH was 5.5. Plants at 22–24 days old were used to measure stomatal aperture. The transgenic line expressing GFP-PATROL1 was grown on solid 1/2 Murashige and Skoog (MS) medium for 7 days in a growth chamber (18/6 h light/dark cycle using white light of 60 μmol m^-2^ s^-1^ at 23.5°C). Cotyledons were used to measure stomatal aperture and GFP-PATROL1 dot densities.

### Stomatal Aperture Measurements

To measure stomatal apertures in response to CO_2_, abaxial epidermal peels were floated on an opening medium containing 10 mM KCl, 25 mM MES-KOH (pH 6.15) and 1 mM CaCl_2_ and incubated in a growth chamber under white light (200 μmol m^-2^ s^-1^) for 1 h. To measure stomatal aperture in response to darkness and ABA, epidermal peels were floated on an opening medium containing 30 mM KCl, 5 mM MES-KOH (pH 6.15) and 1 mM CaCl_2_ and incubated in a growth chamber under white light (120 μmol m^-2^ s^-1^) for 1 h. The epidermal strips were transferred to darkness or the opening medium with or without 2 mM bicarbonate or 10 μM ABA and inhibitors and incubated for a further 2 h before stomatal apertures were measured.

### Measurements of GFP-PATROL1 Dot Density

To evaluate the density of GFP-PATROL1 dots beneath plasma membranes quantitatively, we used transgenic seedlings grown on solid 1/2 MS medium for 7 days in growth chambers at 23.5°C with an 18/6 h light/dark cycle using 60 μmol m^-2^s^-1^ white lights. As a pretreatment, seedlings were immersed into 1.0 mL of opening buffer [30 mM KCl, 0.1 mM CaCl_2_, 10 mM MES-KOH (pH 6.15)] in microtubes for 1 h under white light (100 μmol m^-2^s^-1^). To examine the light/dark response, seedlings were transferred into 1.0 mL of the control solution [opening buffer with 0.1% (v/v) DMSO] or inhibitor solutions (opening buffer with 2.5 μM PAO or 70 μM LY294002) wrapped with or without aluminum foil to shield the solution from light, and placed in a 23.5°C chamber with 100 μmol m^-2^s^-1^ white lights for 2–3 h. To examine the ABA response, seedlings were transferred into 1.0 mL of the control solution or inhibitor solutions with or without 10 μM ABA, and placed in a 23.5°C chamber with 100 μmol m^-2^s^-1^ white lights for 2–3 h. To examine the CO_2_ response, seedlings were transferred into 1.0 mL of the control solution or inhibitor solutions with or without 2 mM CsHCO_3_ (Sigma–Aldrich), and placed in a 23.5°C chamber with 100 μmol m^-2^s^-1^ white lights for 2–3 h. Cesium bicarbonate was used as the source of CO_2_ in all experiments. Cotyledons were mounted on glass slides and observed under a variable-angle epifluorescence microscope (IX-73; Olympus) equipped with a total internal reflection microscopy unit (IX3-RFAEVAW; Olympus) and an electron multiplying charge-coupled device camera head system (ImagEM; Hamamatsu Photonics). Time-sequential images were captured using the ‘Acquire-Stream Acquisition’ feature of MetaMorph software (Molecular Devices) with 300 frames at 100 ms exposure time to obtain the maximum intensity projection images. The numbers of GFP-PATROL1 dots in the maximum intensity projection images were counted using the ‘Process-Find Maxima…’ feature of ImageJ software (Abramoff et al., 2004). Cell areas that were manually segmented were measured using the ‘Analyze-Measure’ feature of ImageJ software, and the GFP-PATROL1 dot densities per unit cell area were calculated.

### Chemicals

PAO (Sigma), LY294002 (2-morpholin-4-yl-8-phenylchromen-4-one) (Tokyo Chemical Industry), LY83583 [6-(phenylamino)-5,8-dihydroquinoline-5,8-dione] (Cayman Chemical Company), brefeldin A ((1*R*,2*E*,6*S*,10*E*,11a*S*,13*S*,14a*R*)-1,13-dihydroxy-6-methyl-1,6,7,8,9,11a,12,13,14,14a-decahydro-4*H*-cyclopenta [*f*]oxacyclotridecin-4-one) (Wako Pure Chemical Industries), W-7 [*N*-(6-aminohexyl)-5-chloro-1-naphthalenesulfonamide hydrochloride] (Wako Pure Chemical Industries), *N*-ethylmaleimide (1-ethylpyrrole-2,5-dione) (Tokyo Chemical Industry), and propyzamide (Wako Pure Chemical Industries) stock solutions (100 mM) were prepared in 100% DMSO.

## Results

### The PI4K Inhibitor Specifically Inhibited CO_2_-Induced Stomatal Closure, Whereas the PI3K Inhibitor Specifically Inhibited Darkness-Induced Stomatal Closure

**FIGURE 1:**
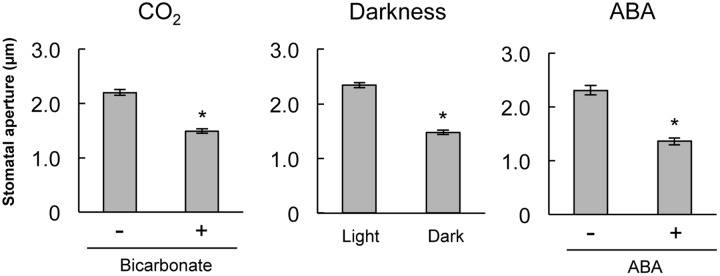
**Stomatal closure induced by bicarbonate, darkness, or abscisic acid (ABA) on stripped epidermal peels of *Arabidopsis thaliana*.** The epidermal strips were placed in opening buffer for 1 h in white light before being transferred to darkness or opening buffer containing 2 mM bicarbonate or 10 μM ABA for 2 h. Error bars indicate ±SE, and asterisks represent a significant difference between treatments (*n* ≥ 120 stomata per each treatment from ten independent experiments; *P* < 0.05, Student’s *t*-test).

To confirm that stomatal guard cells respond to CO_2_, darkness, and ABA treatment, we assayed stomatal closure in epidermal strips of mature *A. thaliana*. Treatments with 2 mM bicarbonate, darkness, and 10 μM ABA led to significant decreases in the average stomatal aperture compared with the control condition (**Figure 1**). This result verifies that guard cells on *A. thaliana* epidermal strips function in response to CO_2_, darkness, and ABA treatment.

**FIGURE 2:**
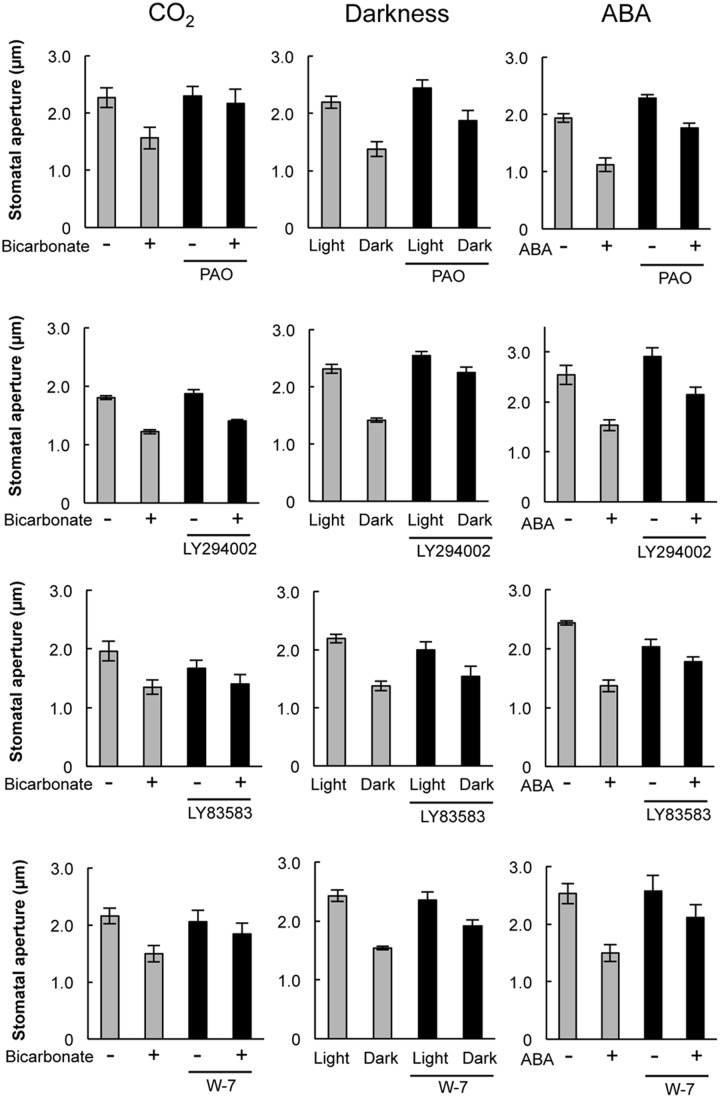
**Effects of membrane trafficking inhibitors on stomatal responses to bicarbonate, darkness, and ABA.** Epidermal strips were placed in opening buffer for 1 h in the light before being transferred to darkness or opening buffer containing 2 mM bicarbonate or 10 μM ABA with/without inhibitors (2.5 μM PAO, 70 μM LY294002, 70 μM LY83583, 70 μM W-7) for 2 h. Error bars indicate ±SE, and lowercase letters represent significantly different groups (*n* ≥ 120 stomata per each treatment from four or five independent experiments; *P* < 0.05, Student’s *t*-test).

**FIGURE 3:**
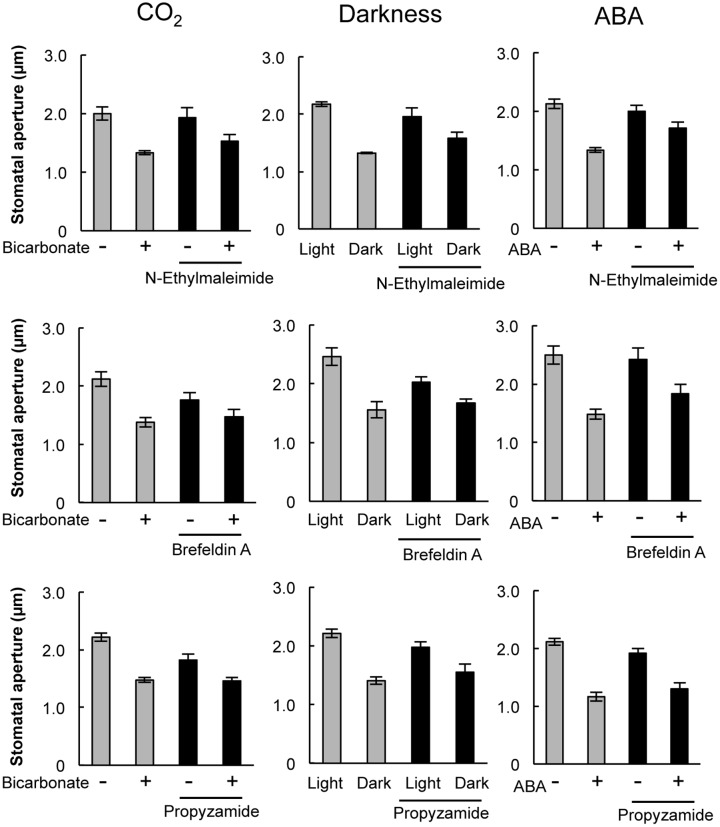
**Effects of membrane trafficking inhibitors on stomatal responses to bicarbonate, darkness, and ABA.** Epidermal strips were placed in opening buffer for 1 h in the light before being transferred to darkness or opening buffer containing 2 mM bicarbonate or 10 μM ABA with/without inhibitors (20 μM *N*-ethylmaleimide, 70 μM brefeldin A, and 80 μM propyzamide) for 2 h. Error bars indicate ±SE, and lowercase letters represent significantly different groups (*n* ≥ 120 stomata per each treatment from four or five independent experiments; *P* < 0.05, Student’s *t*-test).

**FIGURE 4:**
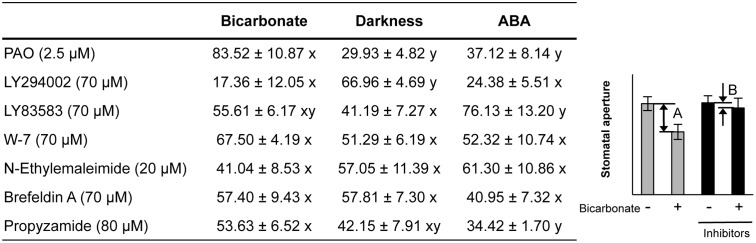
**Effects of membrane trafficking inhibitors on stomatal responses to bicarbonate, darkness, and ABA.** A representative graph of stomatal aperture measurements is given to illustrate the calculation of inhibition levels (right figure inset). Inhibition levels were calculated from the data shown in **Figures 2**, **3** as the percentage difference in stomatal closure with(B)/without(A) the specific inhibitor based on the equation 100 × (1-B/A) (as shown in the figure inset). Values are presented as means ±SE, and lowercase letters represent significantly different groups (*n* ≥ 120 stomata per treatment from three independent experiments; *P* < 0.05, Fisher’s LSD test).

Pharmacological tests to determine whether membrane trafficking is involved in the stomatal closure response to CO_2_, darkness, and ABA used inhibitors of PI4K (PAO), PI3K (LY294002), the cGMP pathway (LY83583), calmodulin (W-7), the SNARE complex (*N*-ethylmaleimide), G-proteins (brefeldin A), and microtubules (propyzamide) (**Table 1** and **Figures 2**, **3**). These inhibitors have been reported previously to inhibit their cellular component targets at the micromolar level. To exclude the possibility of non-specific effects at high inhibitor concentrations, inhibitor concentrations were selected that would lead to one-half inhibition of stomatal aperture changes compared with the control conditions. We defined the inhibition levels as shown in the **Figure 4**, thereby enabling quantitative comparisons of the effect of each inhibitor on the stomatal response (**Figure 4**).

**Table 1:** Membrane trafficking inhibitors used in this study.

Inhibitors	Inhibition targets	Inhibitor effects	Reference
Phenylarsine oxide	PI 4-phosphate (PI4P)	PI4K inhibition	Wiedermann et al., 1996
LY294002	PI3P	PI3K inhibition	Vlahos et al., 1994
LY83583	Cyclic GMP	Guanylyl cyclase inhibition	Mülsch et al., 1988
W-7	Calmodulin	Calmodulin inhibition	Kanamori et al., 1981
*N*-Ethylmaleimide	SNARE complex	SNARE complex dissociation	Smyth, 1969
Brefeldin A	G protein	GNOM ARF-GEF inhibition	Nebenführ et al., 2002
Propyzamide	Microtubule	Microtubule depolymerization	Akashi et al., 1988


When epidermal strips of mature leaves were treated with PAO, a PI4K inhibitor, stomatal apertures were reduced after bicarbonate, darkness, and ABA treatments by 83.5, 29.9, and 37.1%, respectively, compared with the control conditions (**Figures 2**, **4**). A PI3K inhibitor LY294002 inhibited stomatal responses to bicarbonate, darkness, and ABA treatments by 17.4, 67.0, and 24.4%, respectively, compared with the control conditions (**Figures 2**, **4**). PAO had a specific and stronger inhibitory effect on the stomatal response to CO_2_ compared with that of darkness and ABA. LY294002 also had a specific and stronger inhibitory effect on the stomatal response to darkness compared with that of CO_2_ and ABA. A cGMP inhibitor, LY83583, inhibited the stomatal responses to bicarbonate, darkness, and ABA treatments by 55.6, 41.2, and 76.1%, respectively, compared with the control conditions (**Figures 2**, **4**). Treatment with LY83583 caused a slightly stronger inhibitory effect in the stomatal responses to ABA. A calmodulin antagonist, W-7, inhibited the stomatal responses to bicarbonate, darkness, and ABA treatments by 67.5, 51.3, and 52.3%, respectively, compared with the control conditions (**Figures 2**, **4**). An inhibitor of the SNARE complex, *N*-ethylmaleimide, inhibited the stomatal responses to bicarbonate, darkness, and ABA treatments by 41.0, 57.0, and 61.3%, respectively, compared with the control conditions (**Figures 3**, **4**). A small G-protein inhibitor, brefeldin A, inhibited 57.4, 57.8, and 41.0% of stomatal response to bicarbonate, darkness, and ABA treatments, respectively, compared with the control conditions (**Figures 3**, **4**). W-7, *N*-ethylmaleimide, and brefeldin A had no specific inhibitory effects on the stomatal response to CO_2_, darkness, or ABA. An inhibitor of microtubule polymerization, propyzamide, inhibited the stomatal responses to bicarbonate, darkness, and ABA treatments by 53.6, 42.1, and 34.4%, respectively, compared with the control conditions (**Figures 3**, **4**). Propyzamide treatment caused a slight but not significant difference in the inhibition of stomatal responses to CO_2_ and ABA.

### PI4K and PI3K Inhibitors Specifically Inhibited the Increase in GFP-PATROL1 Dot Density in Response to CO_2_ and Darkness, Respectively

Specific inhibitory effects were observed in the stomatal response to CO_2_ and darkness when epidermal strips were treated with PAO and LY294002, respectively (**Figures 2**, **4**). In guard cells, GFP-PATROL1 dot densities increase during stomatal closure (Hashimoto-Sugimoto et al., 2013). To further confirm the effects of PAO and LY294002 on the behavior of PATROL1 in response to CO_2_, darkness, and ABA, we measured GFP-PATROL1 dot density in the guard cells treated with 2 mM bicarbonate, darkness, and 10 μM ABA in the presence of PAO and LY294002. Young seedlings (7-days-old) were used in this experiment because preliminary analyses demonstrated that there is a significant reduction in GFP-PATROL1 fluorescence in the guard cells of mature leaves. Further tests showed that GFP-PATROL1 dots were eliminated with a 70 μM LY294002 treatment. Furthermore, the 30 μM LY294002 treatment was sufficient for inhibiting darkness-induced PATROL1 dynamics under our experimental conditions. Therefore, subsequent experiments used 30 μM LY294002.

**FIGURE 5:**
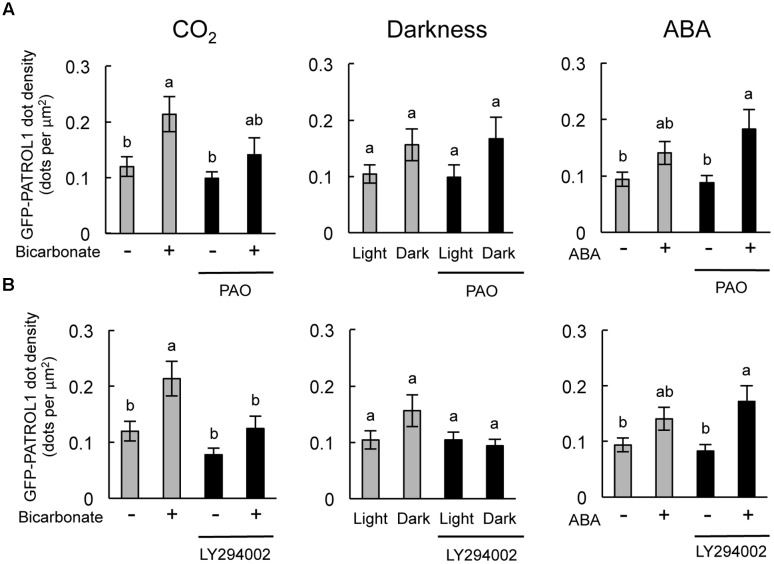
**Effects of PAO and LY294002 on GFP-PATROL1 dot densities in cotyledonary guard cells in response to bicarbonate, darkness, and ABA.** **(A)** Cotyledons were placed in opening buffer for 1 h in white light before being transferred to darkness or opening buffer containing 2 mM bicarbonate or 10 μM ABA with/without 2.5 μM PAO for 2 h. Error bars indicate ±SE of 12 independent experiments, and lowercase letters represent significantly different groups (*P* < 0.05, Mann–Whitney’s *U*-test). **(B)** Cotyledons were placed in opening buffer for 1 h in white light before being transferred to darkness or opening buffer containing 2 mM bicarbonate or 10 μM ABA with/without 30 μM LY294002 for 2 h. Error bars indicate ±SE of 12 independent experiments, and lowercase letters represent significantly different groups (*P* < 0.05, Turkey-kramer’s test).

The stomatal closure assay using cotyledons expressing GFP-PATROL1 showed a similar trend in the responsiveness to CO_2_, darkness, and ABA between cotyledonary leaves and mature leaves when exposed to PAO and LY294002 (Supplementary Figure S1). Time-sequential images of GFP-PATROL1 movement showed that GFP-PATROL1 dot appeared in response to 2 mM bicarbonate, darkness, or 10 μM ABA treatment in guard cells (Supplementary Figures S3, S4) and resulted in significant increases in PATROL1 dot densities (**Figure 5**). These results suggest that activation of PATROL1 dynamics is essential for its function in the environmental signal transduction pathways. Compared with the control condition, PAO treatment significantly inhibited the appearance of GFP-PATROL1 dots (Supplementary Figure S3) and the increase in GFP-PATROL1 dot density in cotyledons after bicarbonate treatment (**Figure 5A**). Treatment with LY294002 also led to a significant inhibition in the appearance of GFP-PATROL1 dots (Supplementary Figure S4) and in the increase in GFP-PATROL1 dot density in cotyledons treated with darkness (**Figure 5B**). Taken together, these results show that PI4K and PI3K may contribute to stomatal closure and PATROL1 dynamics in response to CO_2_ and darkness, respectively.

## Discussion

We used several specific inhibitors to determine how certain cellular components in guard cells contribute to the stomatal closure response to CO_2_, darkness, and ABA. Some previous inhibitor studies (Jung et al., 2002; Dubovskaya et al., 2011; Jiang et al., 2014) determined where the inhibitors act in the ABA signal transduction pathways; however, it remains unknown how these cellular components are involved in the environmental signal transduction pathways. In this study, we show that PI4K and PI3K modulate signal transduction pathways in the stomatal response to CO_2_ and darkness, respectively. The concentrations of inhibitors used in this study were chosen to not inhibit the stomatal closure response completely, but to enable a comprehensive investigation of the inhibitory effects on the stomatal response to CO_2_, darkness, and ABA. PIs in guard cells are reported to be involved in several aspects of stomatal movement. PI4P and PI3P mediate [Ca^2+^]_cyt_ increase in response to ABA (Jung et al., 2002). PI(3,5)P_2_ is required for acidification of vacuoles in *Vicia faba* guard cells during ABA-induced stomatal closure (Bak et al., 2013). In addition, PI(4,5)P_2_ is important for light-induced stomatal opening (Lee et al., 2007) and ABA-induced closing responses (Jacob et al., 1999). These results imply that PIs affect not only the known regulatory mechanisms for stomatal movements but also the complex networks of environmental signal transduction. We propose that our study also provides new mechanistic insight into the sensing and transduction pathways of environmental signals.

We observed similar levels of stomatal closure inhibition among bicarbonate, darkness, and ABA when epidermal strips were treated with W-7, brefeldin A, and *N*-ethylmaleimide (**Figure 4**). These results implied that calmodulin, cGMP, and the SNARE complex are involved in stomatal closure signaling irrespective of environmental factors. These components might act in a downstream signaling network that is activated by various environmental signals. Alternatively, there may be non-specific effects of these inhibitors, even when using concentrations that inhibit changes in stomatal closure by one-half compared with the control condition. LY83583 inhibited stomatal closure in response to ABA stronger than the response to darkness (**Figure 4**), implying that cGMP is more associated with ABA signaling than the environmental signal transduction pathways. This finding is consistent with a report that cGMP acts downstream of H_2_O_2_ and NO in the signaling pathway by which ABA induces stomatal closure (Dubovskaya et al., 2011). We also observed a stronger inhibition of stomatal response to CO_2_ compared with ABA when epidermal strips were exposed to propyzamide (**Figure 4**). Propyzamide inhibits stomatal opening induced by light (Marcus et al., 2001) but does not inhibit stomatal closure induced by ABA (Jiang et al., 2014). These results imply that microtubules are also involved in the environmental signal transduction pathway for stomatal responses.

Previously, Higaki et al. (2014) found that 20 μM LY294002 did not perturb the appearance of GFP-PATROL1 dots, whereas the GFP-PATROL1 dots disappeared with 20 μM PAO treatment. In this study, 20 μM LY294002 did not affect stomatal closure in response to CO_2_, darkness, and ABA (Supplementary Figure S2 nor the activity of GFP-PATROL1. However, the GFP-PATROL1 dot densities as well as stomatal movements were inhibited by 2.5 μM PAO and 30 μM LY294002 (**Figure 5**). These observations indicate that PI4K and PI3K also regulate PATROL1 dynamics. Recently, PI4K and PI3K were reported to have distinct roles in intracellular trafficking of cellulose synthase complexes (CESA) (Fujimoto et al., 2015). PI4K is required for the internalization of GFP-CESA3 from the plasma membrane to Golgi apparatus. In contrast, PI3K has a role in secretion and/or recycling to the plasma membrane (Fujimoto et al., 2015). Although it is not clear whether the role is direct or indirect, the reduced PATROL1 dynamics after PAO and LY294002 treatment may reflect the existence of unknown mechanisms involving PI4K and PI3K in the differential regulation of PATROL1 dynamics. In this study, we showed that PI4K and PI3K are involved in the intermediate steps between the sensing of environmental signals and membrane trafficking systems. Further studies will be needed to elucidate mechanisms of PI metabolism for sensing of environmental signals; such studies will also be effective in elucidating the possible coordination between PI metabolism and membrane trafficking systems.

## Author Contributions

ST, KM, and KI conceived the ideas for the study. All the authors participated in data analysis and interpretation, and contributed to the writing and editing of the manuscript. ST, KM, and TH contributed to the experimentation.

## Conflict of Interest Statement

The authors declare that the research was conducted in the absence of any commercial or financial relationships that could be construed as a potential conflict of interest. The reviewer WY declared a shared affiliation, though no other collaboration, with several of the authors TH, SH to the handling Editor, who ensured that the process nevertheless met the standards of a fair and objective review.

## References

[B1] Abramoff M. D. Magelhaes P. J. Ram S. J. (2004). Image processing with Image J. *Biophoton. Int.* 11 36–42.

[B2] Akashi T. Izumi K. Nagano E. Enomoto M. Mizuno K. Shibaoka H. (1988). Effects of propyzamide on tobacco cell microtubules *in vivo* and *in vitro*. *Plant Cell Physiol.* 29 1053–1062.

[B3] Assmann S. M. Jegla T. (2016). Guard cell sensory systems: recent insights on stomatal responses to light, abscisic acid, and CO2. *Curr. Opin. Plant Biol.* 33 157–167. 10.1016/j.pbi.2016.07.003 27518594

[B4] Bak G. Lee E. J. Lee Y. Kato M. Segami S. Sze H. (2013). Rapid structural changes and acidification of guard cell vacuoles during stomatal closure require phosphatidylinositol 3,5-bisphosphate. *Plant Cell* 25 2202–2216. 10.1105/tpc.113.110411 23757398 PMC3723621

[B5] Dubovskaya L. V. Bakakina Y. S. Kolesneva E. V. Sodel D. L. McAinsh M. R. Hetherington A. M., (2011). cGMP-dependent ABA-induced stomatal closure in the ABA-insensitive Arabidopsis mutant *abi1-1*. *New Phytol.* 191 57–69. 10.1111/j.1469-8137.2011.03661.x 21371039

[B6] Eisenach C. Chen Z. H. Grefen C. Blatt M. R. (2012). The trafficking protein SYP121 of Arabidopsis connects programmed stomatal closure and K+ channel activity with vegetative growth. *Plant J.* 69 241–251. 10.1111/j.1365-313X.2011.04786.x 21914010

[B7] Fujimoto M. Suda Y. Vernhettes S. Nakano A. Ueda T. (2015). Phosphatidylinositol 3-kinase and 4-kinase have distinct roles in intracellular trafficking of cellulose synthase complex in *Arabidopsis thaliana*. *Plant Cell Physiol.* 59 287–298. 10.1093/pcp/pcu195 25516570

[B8] Garcia-Mata C. Robert G. Sokolovski S. Hills A. Lamattina L. Blatt M. R. (2003). Nitric oxide regulates K+ and Cl- channels in guard cells through a subset of abscisic acid-evoked signaling pathways. *Proc. Natl. Acad. Sci. U.S.A.* 100 11116–11121. 10.1073/pnas.1434381100 12949257 PMC196936

[B9] Hashimoto-Sugimoto M. Higaki T. Yaeno T. Nagami A. Irie M. Fujimi M. (2013). A Munc13-like protein in *Arabidopsis* mediates H^+^-ATPase translocation that is essential for stomatal responses. *Nat. Commun.* 4:2215 10.1038/ncomms3215 PMC3731666 23896897

[B10] Higaki T. Hashimoto-Sugimoto M. Akita K. Iba K. Hasezawa S. (2014). Dynamics and environmental responses of PATROL1 in Arabidopsis subsidiary cells. *Plant Cell Physiol.* 55 773–780. 10.1093/pcp/pct151 24163289

[B11] Jacob T. Ritchie S. Assmann S. M. Gilroy S. (1999). Abscisic acid signal transduction in guard cells is mediated by phospholipase D activity. *Proc. Natl. Acad. Sci. U.S.A.* 96 12192–12197. 10.1073/pnas.96.21.12192 10518598 PMC18434

[B12] Jiang Y. Wu K. Lin F. Qu Y. Liu X. Zhang Q. (2014). Phosphatidic acid integrates calcium signaling and microtubule dynamics into regulating ABA-induced stomatal closure in *Arabidopsis*. *Planta* 239 565–575. 10.1007/s00425-013-1999-5 24271006

[B13] Jung J. Y. Kim Y. W. Kwak J. M. Hwang J. U. Young J. Schroeder J. I. (2002). Phosphatidylinositol 3- and 4-phosphate are required for normal stomatal movements. *Plant Cell* 14 2399–2412. 10.1105/tpc.004143 12368494 PMC151225

[B14] Kanamori M. Naka M. Asano M. Hidaka H. (1981). Effects of N-(6-aminohexyl)-5-chloro-1-naphthalenesulfonamide and other calmodulin antagonists (calmodulin interacting agents) on calcium-induced contraction of rabbit aortic strips. *J. Pharmacol. Exp. Ther.* 217 494–499. 6453220

[B15] Kollist H. Nuhkat M. Roelfsema M. R. G. (2014). Closing gap: linking elements that control stomatal movement. *New Phytol.* 203 44–62. 10.1111/nph.12832 24800691

[B16] Lee Y. Kim Y. W. Jeon B. W. Park K. Y. Suh S. J. Seo J. (2007). Phosphatidylinositol 4,5-bisphosphate is important for stomatal opening. *Plant J.* 52 803–816. 10.1111/j.1365-313X.2007.03277.x 17883374

[B17] Leyman B. Geelen D. Quintero F. J. Blatt M. R. (1999). A tobacco syntaxin with a role in hormonal control of guard cell ion channels. *Science* 283 537–540. 10.1126/science.283.5401.537 9915701

[B18] Marcus A. I. Moore R. C. Cyr R. J. (2001). The role of microtubules in guard cell function. *Plant Physiol.* 125 387–395. 10.1104/pp.125.1.387 11154346 PMC61019

[B19] Mülsch A. Busse R. Liebau S. Förstermann U. (1988). LY83583 interferes with the release of endothelium-derived relaxing factor and inhibits soluble guanylate cyclase. *J. Pharmacol. Exp. Ther.* 247 283–288. 2902213

[B20] Nebenführ A. Ritzenthaler C. Robinson D. G. (2002). Brefeldin A: deciphering an enigmatic inhibitor of secretion. *Plant Physiol.* 130 1102–1108. 10.1104/pp.011569 12427977 PMC1540261

[B21] Neill S. J. Desikan R. Clarke A. Hancock J. T. (2002). Nitric oxide is a novel component of abscisic acid signaling in stomatal guard cells. *Plant Physiol.* 128 13–16. 10.1104/pp.010707 11788747 PMC1540198

[B22] Schroedar J. I. Allen G. J. Hugouvieux V. Kwak J. M. Waner D. (2001). Guard cell signal transduction. *Annu. Rev. Plant Physiol. Plant Mol. Biol.* 52 627–658. 10.1146/annurev.arplant.52.1.627 11337411

[B23] Smyth D. G. (1969). Reactions of *N*-ethylmaleimide with peptides and amino acids. *Biochem. J.* 91 589–595. 10.1042/bj0910589 PMC1202996 5840721

[B24] Staxen I. Pical C. Montgomery L. T. Gray J. E. Hetherington A. M. McAinsh M. R. (1999). Abscisic acid induces oscillations in guard-cell cytosolic free calcium that involve phosphoinositide-specific phospholipase C. *Proc. Natl. Acad. Sci. U.S.A.* 16 1779–1784. 10.1073/pnas.96.4.1779 PMC15593 9990101

[B25] Sutter J. U. Sieben C. Hartel A. Eisenach C. Thiel G. Blatt M. R. (2007). Abscisic acid triggers the endocytosis of the *Arabidopsis* KAT1 K^+^ channel and its recycling to the plasma membrane. *Curr. Biol.* 17 1396–1402. 10.1016/j.cub.2007.07.020 17683934

[B26] Vlahos C. J. Matter W. F. Hui K. Y. Brown R. F. (1994). A specific inhibitor of phosphatidylinositol 3-kinase, 2-(4-morpholinyl)-8-phenyl-4H-1-benzopyran-4-one (LY294002). *J. Biol. Chem.* 269 5241–5248. 8106507

[B27] Wiedermann C. Schäfer T. Burger M. M. (1996). Chromaffin granule-associated phosphatidylinositol 4-kinase activity is required for stimulated secretion. *EMBO J.* 15 2094–2101. 8641275 PMC450131

