# Supplementary material for: Differential Effects of Phosphatidylinositol 4-Kinase (PI4K) and 3-Kinase (PI3K) Inhibitors on Stomatal Responses to Environmental Signals
Source: Front Plant Sci. 2017 May 1;8:677. doi: 10.3389/fpls.2017.00677 (PMC5410623; doi:10.3389/fpls.2017.00677)
Supplement: Supplementary file 1 [file Image_1.pdf]

Supplementary Material

Differential Effects of Phosphatidylinositol 4-Kinase (PI4K) and 3-Kinase (PI3K) Inhibitors on Stomatal Responses to Environmental Signals

Sho Takahashi[1], Keina Monda[1], Takumi Higaki[2], Mimi Hashimoto-Sugimoto[3], Juntaro Negi[1], Seiichiro Hasezawa[2], Koh Iba[1*]

*** Correspondence:** Koh Iba: iba.koh.727@m.kyushu-u.ac.jp

Supplementary Figures

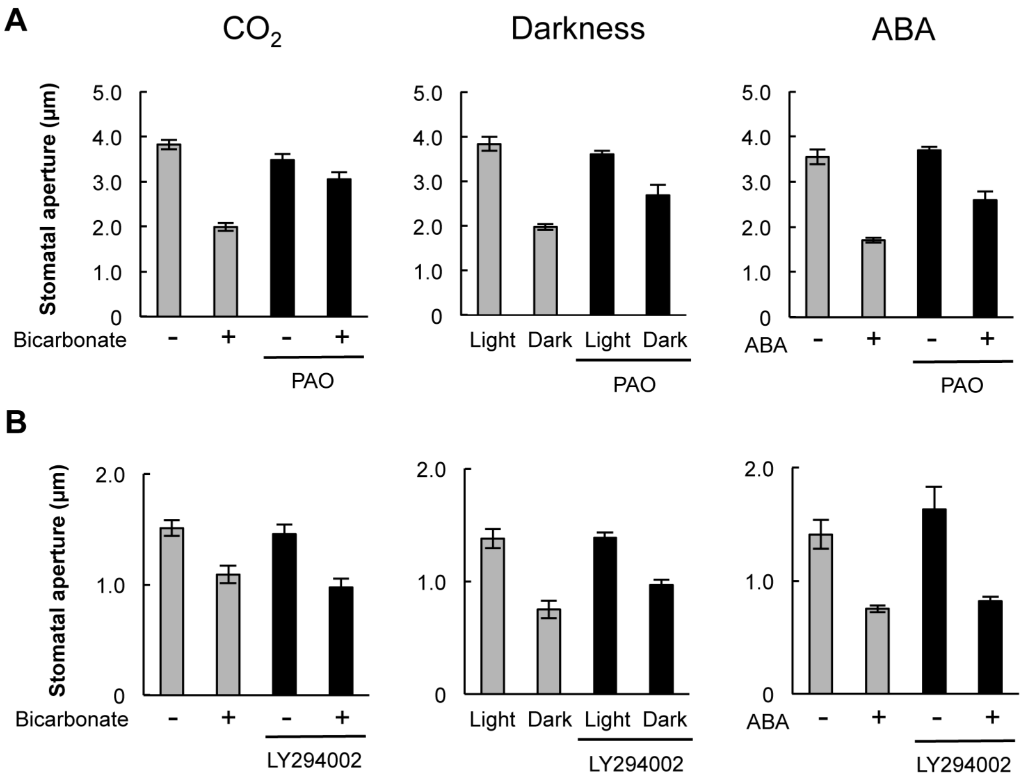

C

	Bicarbonate	Darkness	ABA
PAO (2.5 μM)	77.11 ± 1.00 x	49.31 ± 10.40 y	39.15 ± 11.01 y
LY294002 (30 μM)	0 x	33.15 ± 3.07 y	0 x

Supplementary Figure 1. Effects of 2.5 µM PAO and 30 µM LY294002 on the stomatal response to bicarbonate, darkness, and ABA in cotyledonary leaves and the percentage of inhibition. (A) Effects of 2.5 µM PAO on the stomatal response to bicarbonate, darkness, and ABA. Epidermal strips were placed in opening buffer for 1 h before transfer to darkness or to opening buffer containing 2 mM bicarbonate or 10 µM ABA with/without inhibitors for 2 h. Error bars indicate means ± SE, and lowercase letters represent significantly different groups ($n \geq 120$ stomata per each treatment from three independent experiments). **(B)** Effects of 30 µM LY294002 on the stomatal response to bicarbonate, darkness, and ABA. Epidermal strips were placed in opening buffer for 1 h before transfer to darkness or to opening buffer containing 2 mM bicarbonate or 10 µM ABA with/without inhibitors for 2 h. Error bars indicate means ± SE, and lowercase letters represent significantly different groups ($n \geq 120$ stomata per each treatment from three independent experiments). **(C)** Inhibition levels were calculated as the percentage difference in stomatal closure with(B)/without(A) the specific inhibitor based on the equation $100 \times (1 - B/A)$ (as shown in the Fig 4 inset). Values are presented as means ± SE, and lowercase letters represent significantly different groups ($n \geq 120$ stomata per treatment from three independent experiments; $P < 0.05$, Fisher's LSD test).

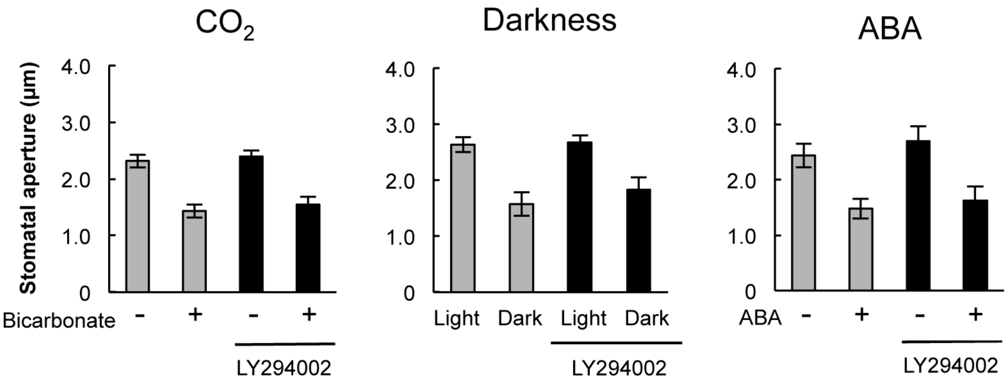

Supplemental Figure 2. Effects of 20 µM LY294002 on the stomatal response to bicarbonate, darkness, and ABA. Epidermal strips were placed in opening buffer for 1 h before transfer to darkness or to opening buffer containing 2 mM bicarbonate or 10 µM ABA with/without inhibitors for 2 h. Error bars indicate means ± SE, and lowercase letters represent significantly different groups (n ≥ 80 stomata per treatment from three independent experiments).

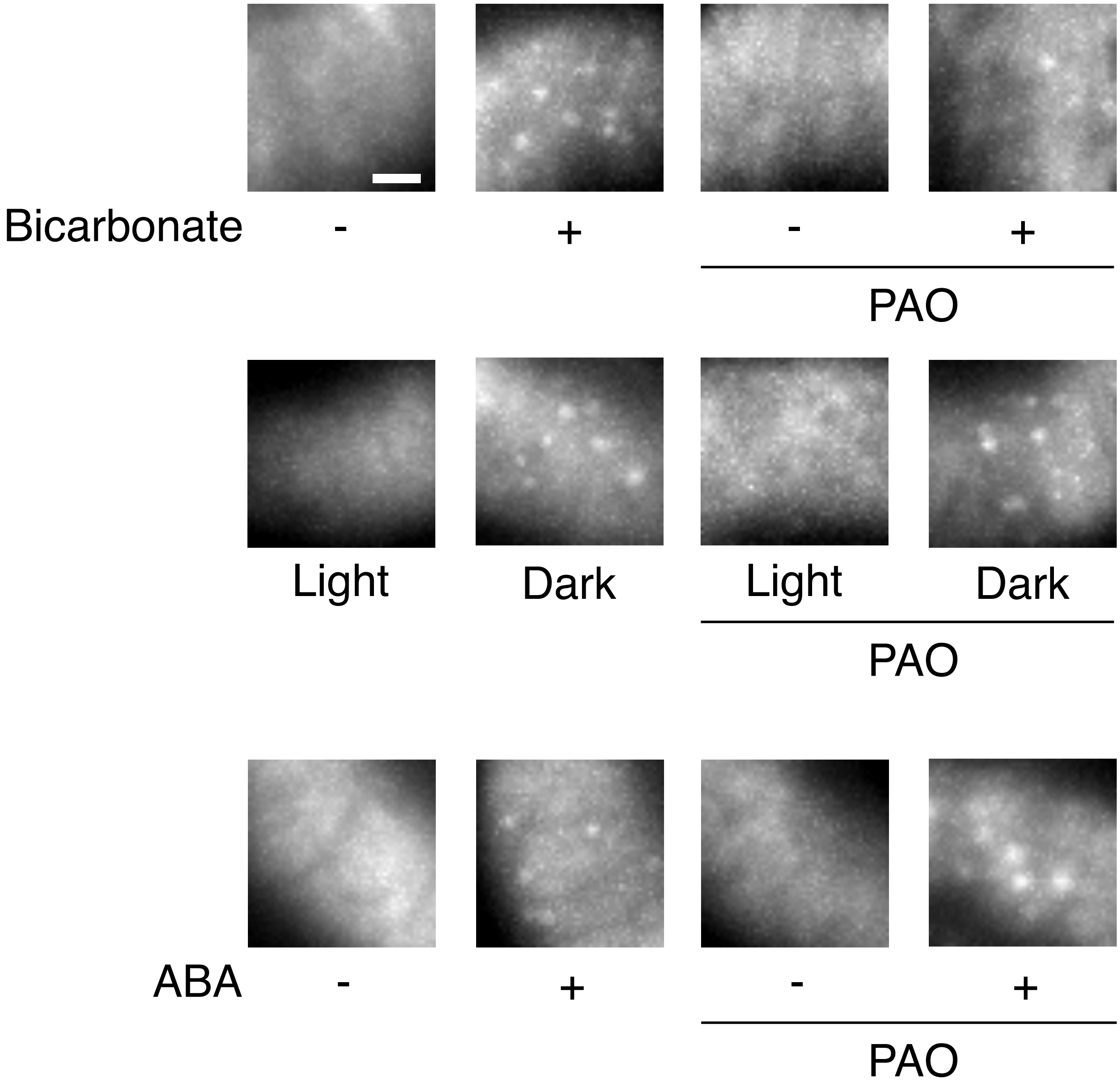

Figure S3. Localization changes of GFP-PATROL1 dots in response to PAO. A single snapshot from time-sequential variable-angle epifluorescence microscope images of GFP-PATROL1 in cotyledonary guard cells. Cotyledons were placed in opening buffer for 1 h in white light before being transferred to darkness or opening buffer containing 2 mM bicarbonate or 10 µM ABA with/without 2.5 µM PAO for 2 h.

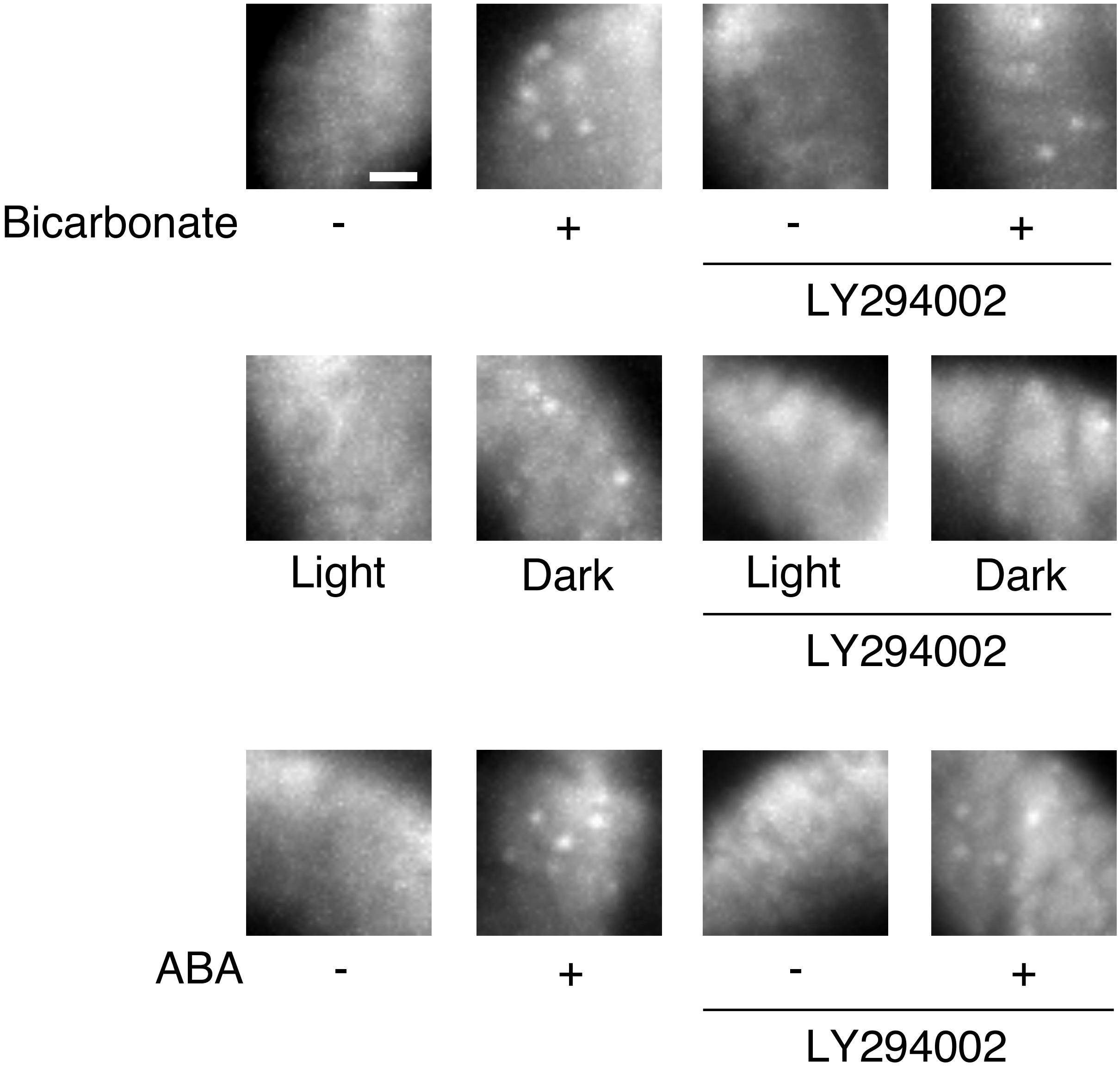

Figure S4. Localization changes of GFP-PATROL1 dots in response to LY294002. A single snapshot from time-sequential variable-angle epifluorescence microscope images of GFP-PATROL1 in cotyledonary guard cells. Cotyledons were placed in opening buffer for 1 h in white light before being transferred to darkness or opening buffer containing 2 mM bicarbonate or 10 μM ABA with/without 30 μM LY294002 for 2 h.
